# Supplementary material for: Development and Validation of a Real-Time Service Model for Noise Removal and Arrhythmia Classification Using Electrocardiogram Signals
Source: Sensors (Basel). 2024 Aug 12;24(16):5222. doi: 10.3390/s24165222 (PMC11360629; doi:10.3390/s24165222)
Supplement: Supplementary file 1 [file sensors-24-05222-s001.zip › sensors-3053071-supplementary.pdf]

Supplementary materials

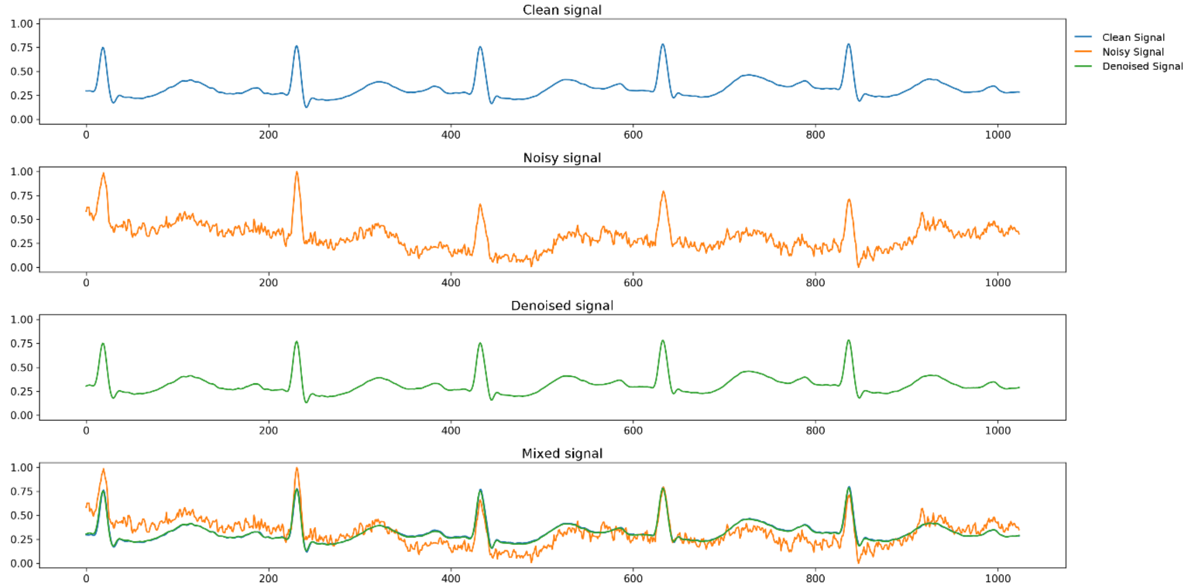

Figure S1. Denoised results of MIT-BIH ECG waveform with added BW noise. Blue, orange, and green colors correspond to the clean, noisy, and denoised signals, respectively. Noise contains 0-dB standard.

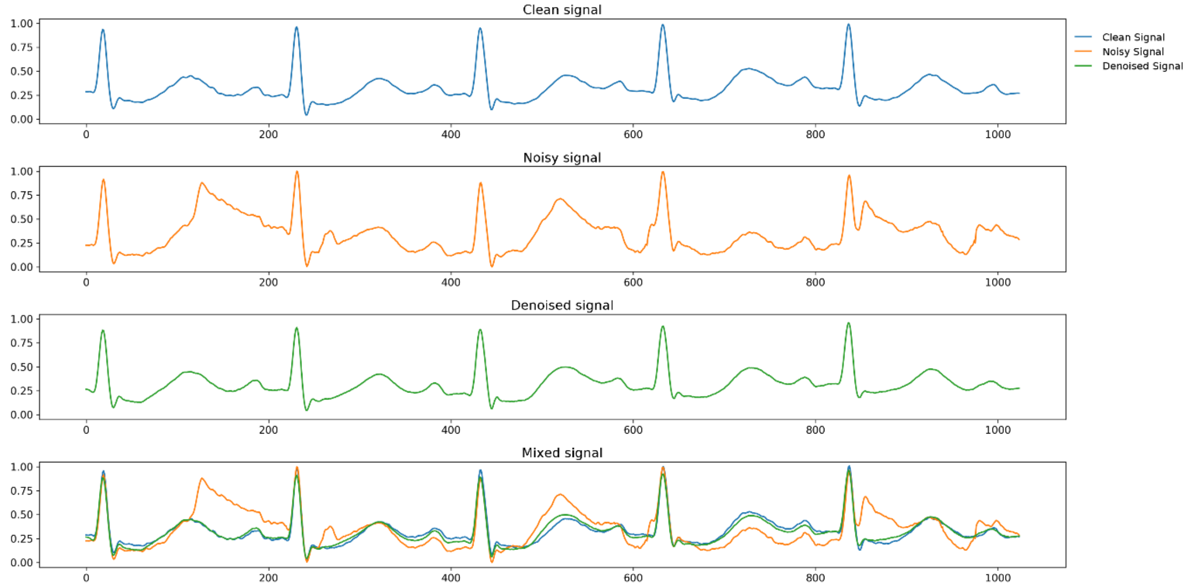

Figure S2. Denoised results of MIT-BIH ECG waveform with added EM noise. Blue, orange, and green colors correspond to clean, noisy, and denoised signals, respectively. Noise contains 0-dB standard.

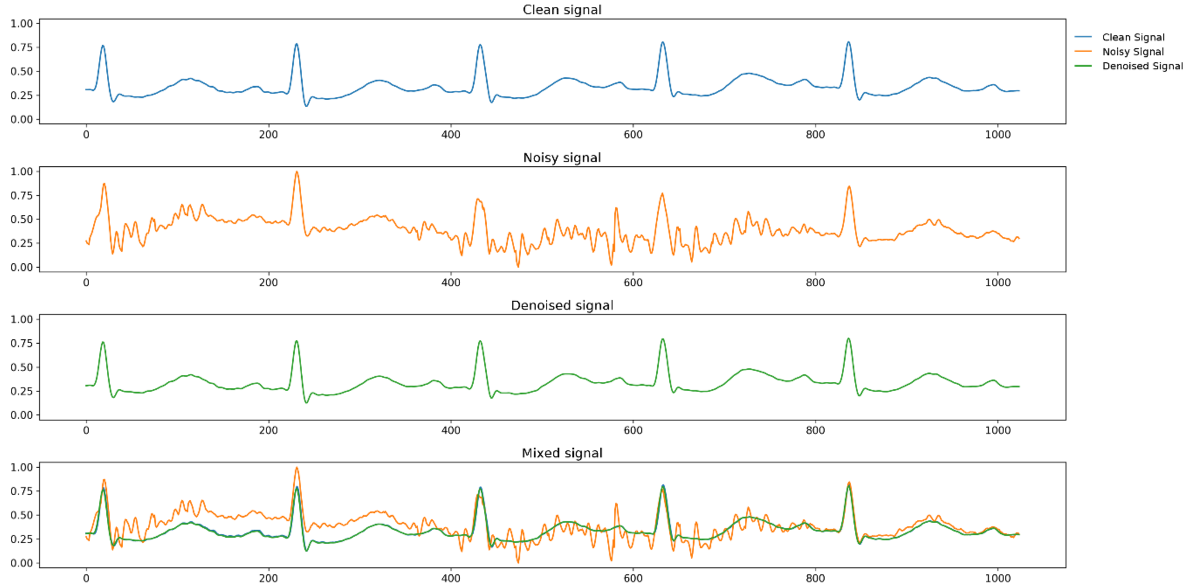

Figure S3. Denoised results of MIT-BIH ECG waveform with added MA noise. Blue, orange, and green colors correspond to clean, noisy, and denoised signals, respectively. Noise contains 0-dB standard.

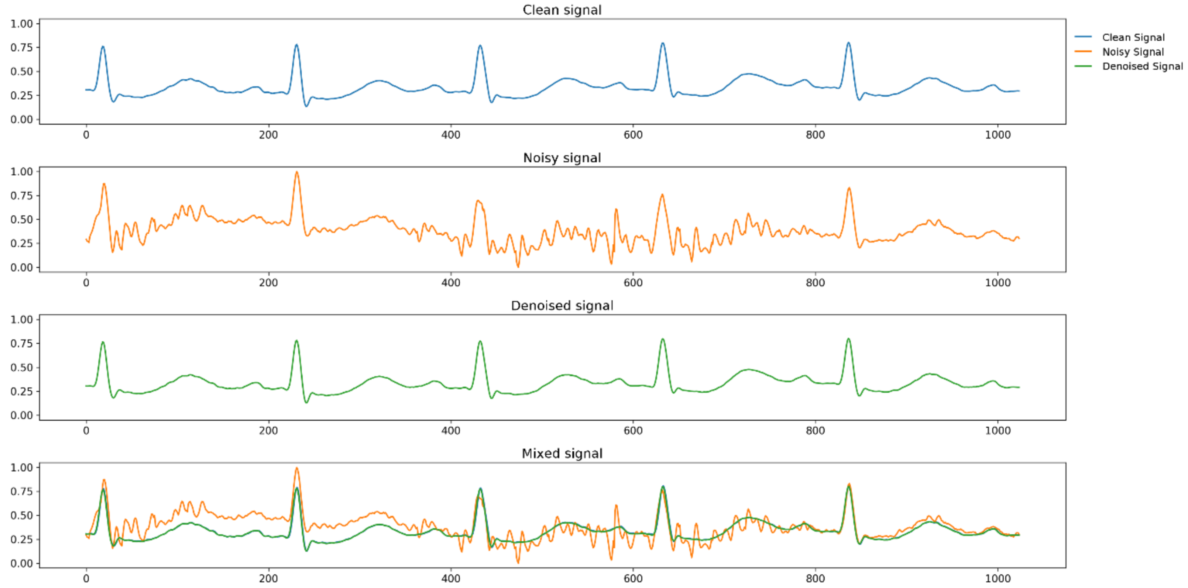

Figure S4. Denoised results of MIT-BIH ECG waveform with added 0.3·BW + 0.7·MA noise. Blue, orange, and green colors correspond to clean, orange, and green signals, respectively. Noise contains 0-dB standard.

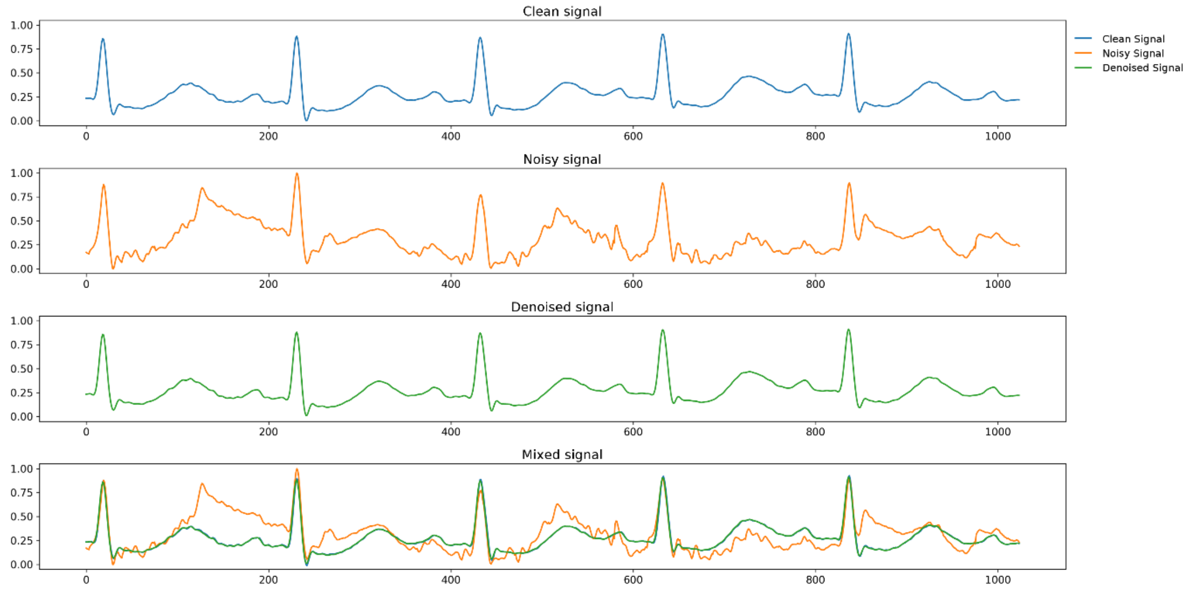

Figure S5. Denoised results of MIT-BIH ECG waveform with added 0.3·EM + 0.7·MA noise. Blue, orange, and green colors correspond to clean, noisy, and denoised signals, respectively. Noise contains 0-dB standard.

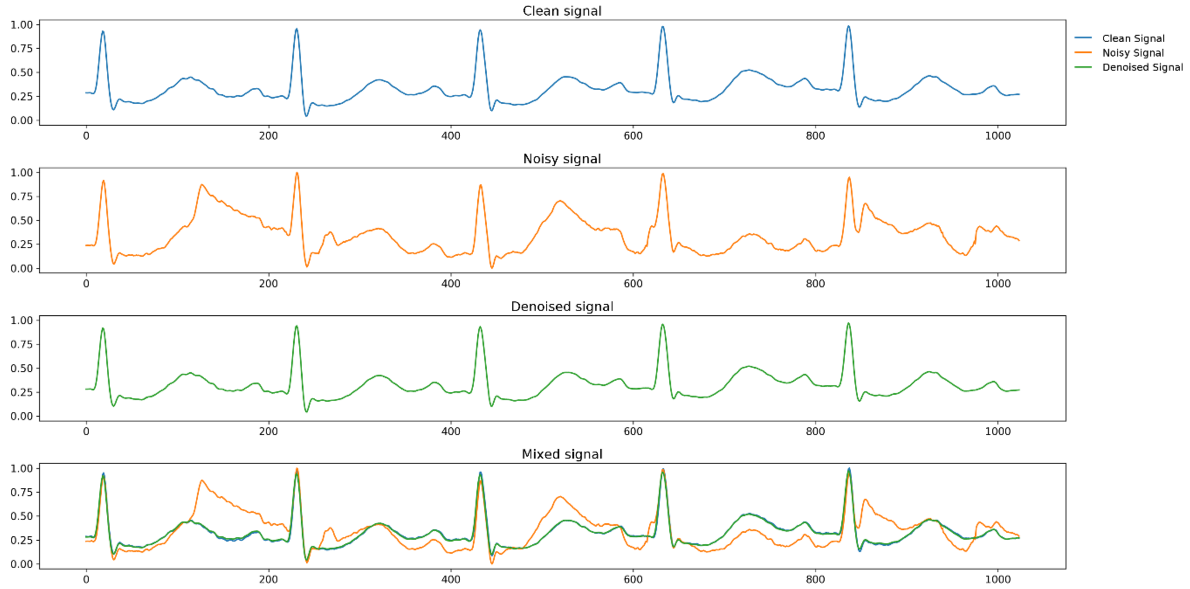

Figure S6. Denoised results of MIT-BIH ECG waveform with added 0.5·BW + 0.5·EM noise. Blue, orange, and green colors correspond to clean, noisy, and denoised signals, respectively. Noise contains 0-dB standard.

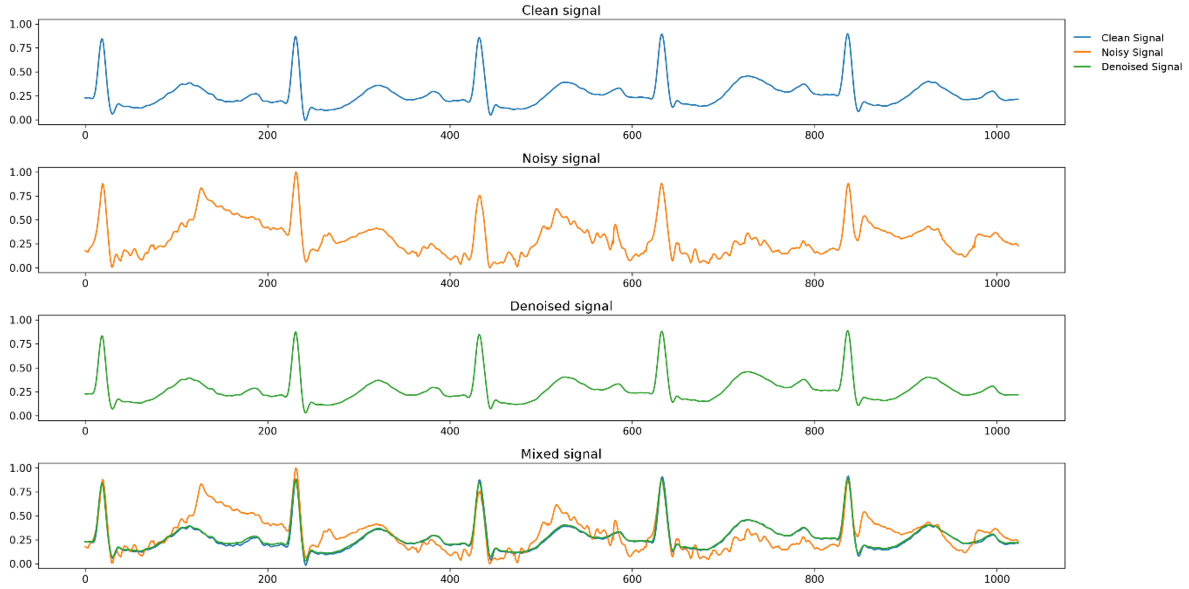

Figure S7. Denoised results of MIT-BIH ECG waveform with added 0.25·BW + 0.25·EM + 0.5·MA noise. Blue, orange, and green colors correspond to clean, noisy, and denoised signals, respectively. Noise contains 0-dB standard.

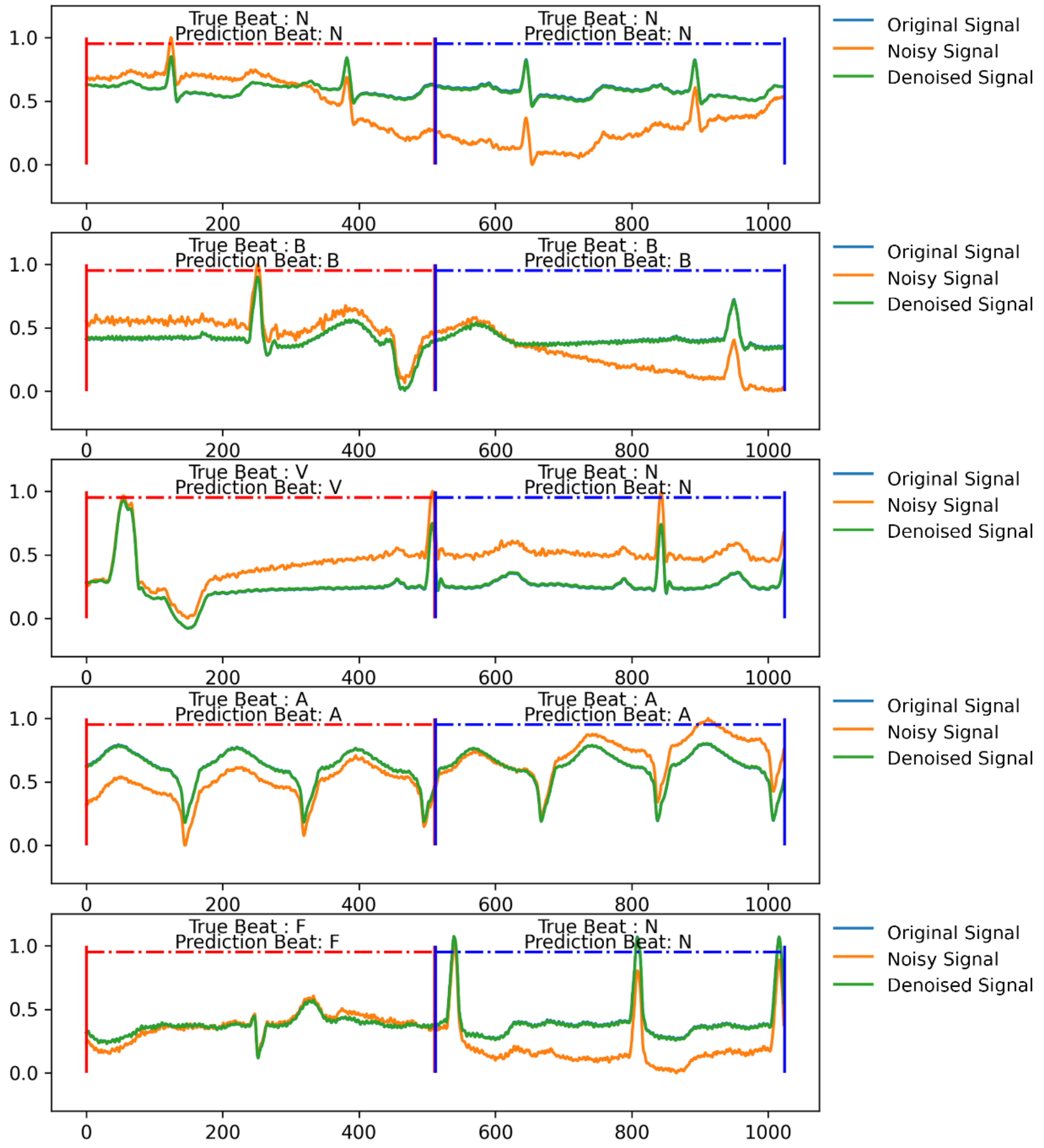

Figure S8. Classification results of MIT-BIH ECG waveform with added BW noise. Classification corresponds to the result for the green denoised signal. Such predictions correspond to each type of true beat. Red wire indicates 0–512 Hz noise, and blue wire indicates 512–1024 Hz noise. True Beat and Prediction Beat are actual and predicted beat values, respectively, for each section. Noise contains 0-dB standard, and from the top, it is composed in order of normal beat (N), B: interventricular block, interventricular block (B) premature ventricular contraction (V), atrial premature beat (A), and fusion of ventricles and normal beats (/).

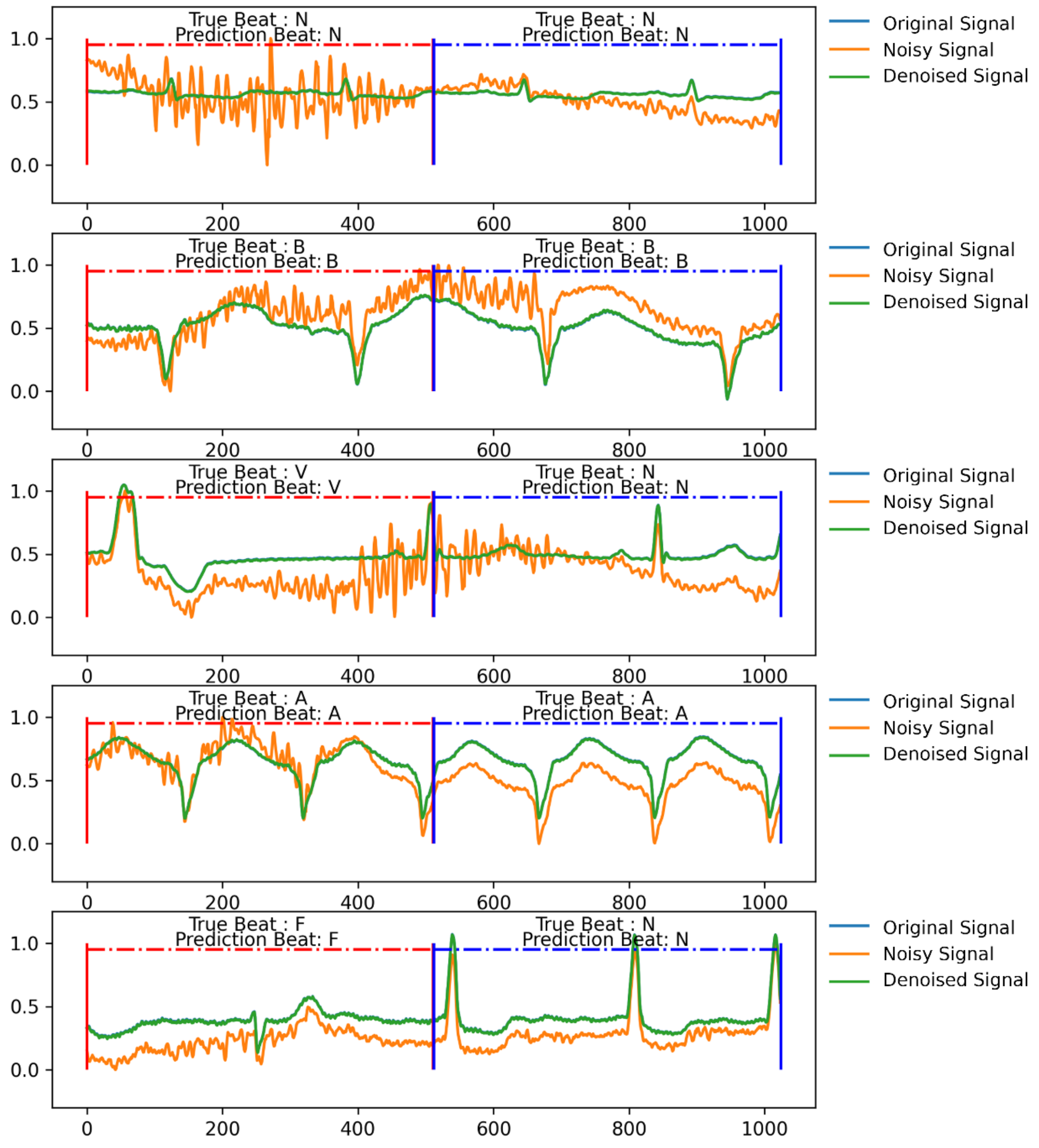

Figure S9. Classification results of MIT-BIH ECG waveform with added EM noise. Classification corresponds to the result of the green denoised signal. Such predictions correspond to each type of true beat. Red wire represents 0–512 Hz, and blue wire represents 512–1024 Hz. True and Prediction Beats are actual and predicted beat values for each section, respectively. Noise contains 0 dB standard, and from the top, it is composed in order of normal beat (N), interventricular block (B), premature ventricular contraction (V), atrial premature beat (A), and fusion of ventricles and normal beats (/).

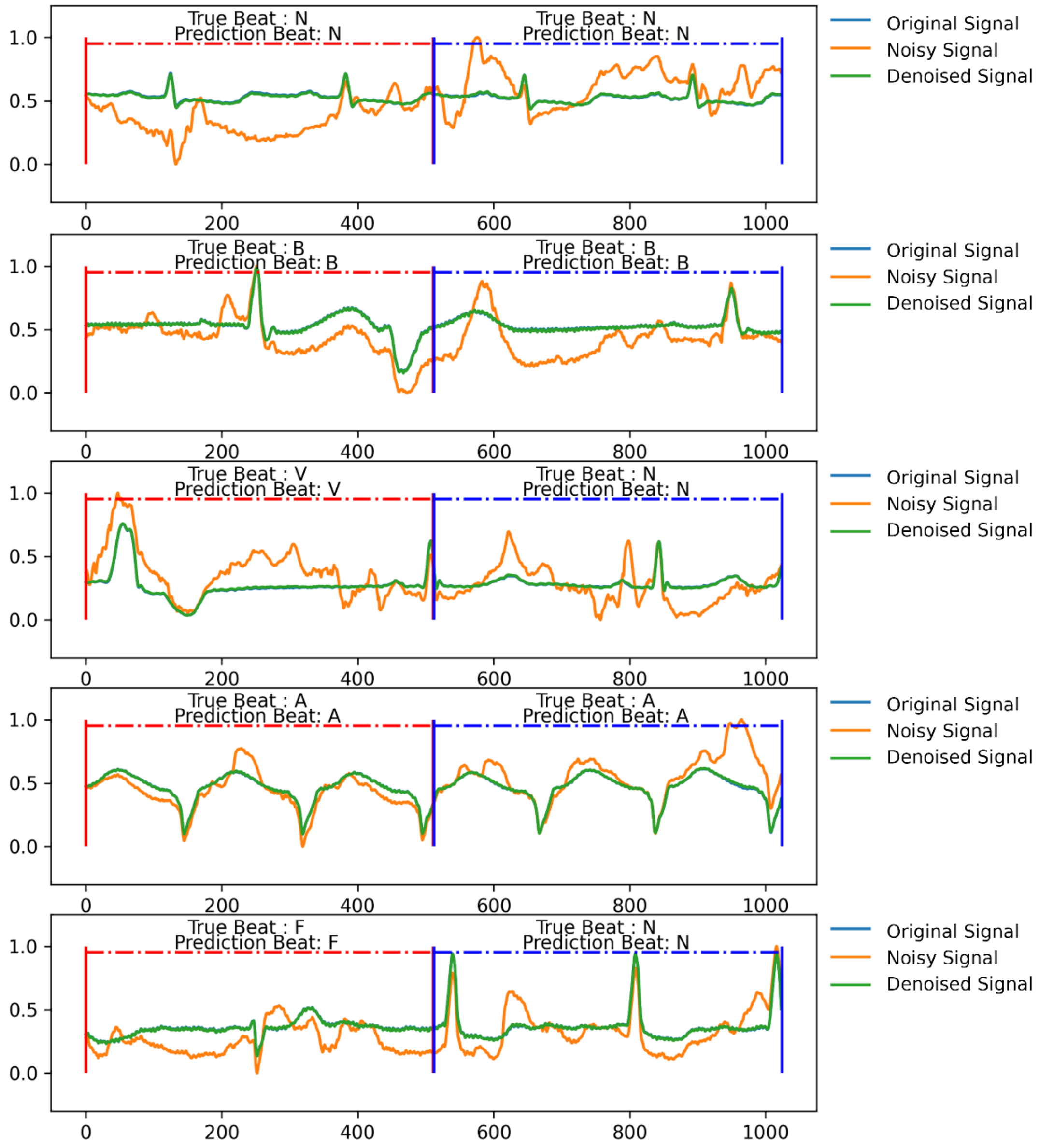

Figure S10. Classification results of MIT-BIH ECG waveform with added MA noise. Classification corresponds to the result of the green denoised signal. Such predictions correspond to each type of true beat. Red and blue wires indicate 0–512 and 512–1024 Hz noise, respectively. True and Prediction Beats are actual and predicted beat values for each section, respectively. Noise contains 0 dB standard, and from the top, it is composed in order of normal beat (N), interventricular block (B), premature ventricular contraction (V), atrial premature beat (A), and fusion of ventricles and normal beats (/).

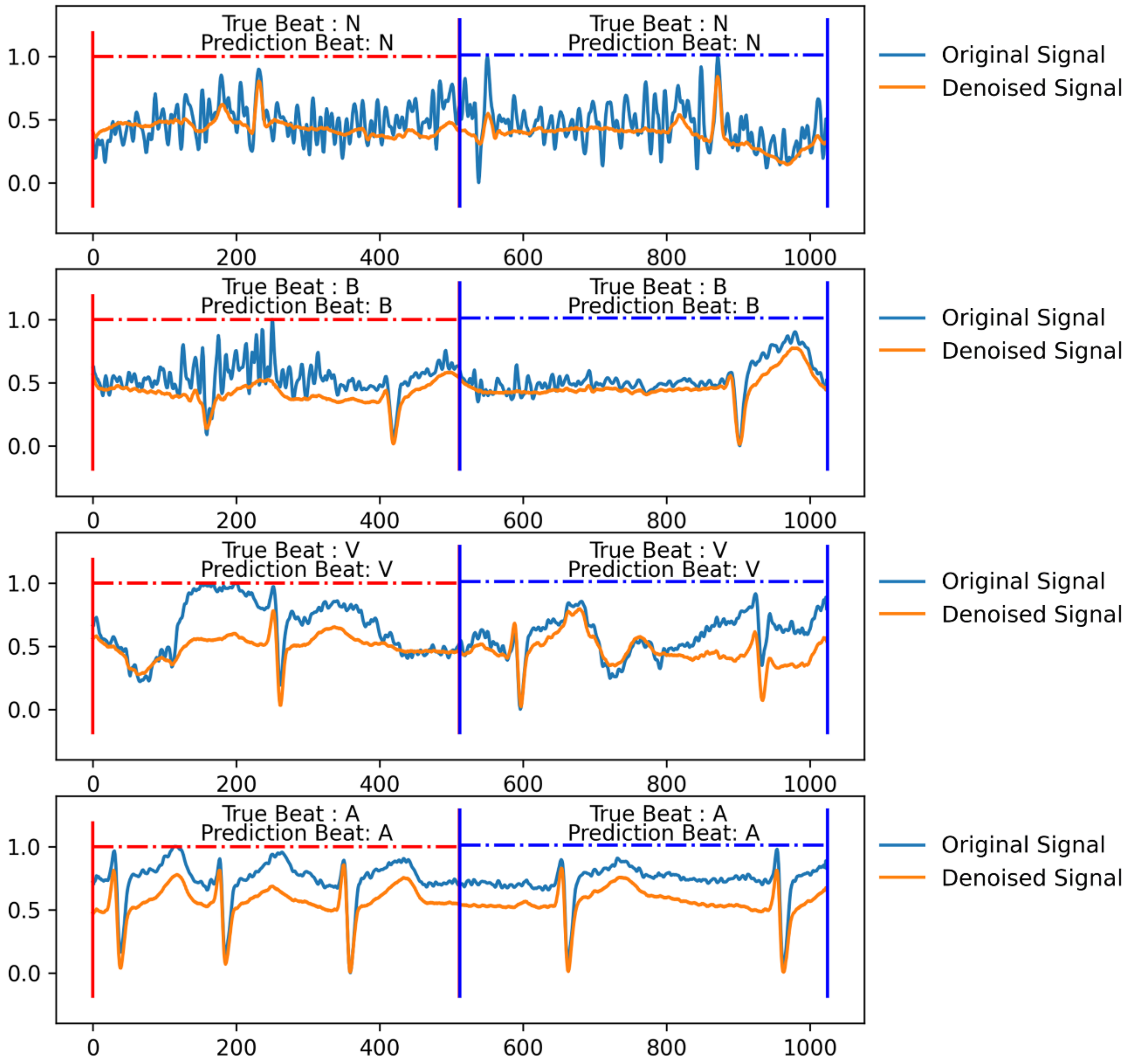

Figure S11. Classification results of measured ECG waveform. Classification corresponds to the result of the green denoised signal. Such predictions correspond to each type of true beat. Red and blue wires indicate 0–512 and 512–1024 Hz noise, respectively. True and Prediction Beats are actual and predicted beat values, respectively, for each section. From the top, it is composed in order of normal beat (N), interventricular block (B), premature ventricular contraction (V), and atrial premature beat (A).
